# Hereditary diffuse gastric cancer in progress: Comparative lessons from Lynch syndrome

**DOI:** 10.1038/s41431-025-01992-w

**Published:** 2025-12-22

**Authors:** Joana Pereira, Luísa Carvalho, Soraia Melo, Patrícia Carneiro, Maria Sofia Fernandes, Raquel Seruca, Joana Figueiredo

**Affiliations:** 1 i3S - Instituto de Investigação e Inovação em Saúde, University of Porto, R. Alfredo Allen 208, 4200-135 Porto, Portugal; 2 Institute of Molecular Pathology and Immunology of the University of Porto (IPATIMUP), R. Júlio Amaral de Carvalho 45, 4200-135 Porto, Portugal; 3 Medical Faculty of the University of Porto, Alameda Prof. Hernâni Monteiro, 4200-319 Porto, Portugal; 4 Institute for Systems and Robotics (ISR/IST), LARSyS, Instituto Superior Técnico, Av. Rovisco Pais 1, 1049-001 Lisboa, Portugal

**Keywords:** Gastric cancer, Cancer genetics

## Abstract

Hereditary diffuse gastric cancer (HDGC) and Lynch syndromes are dominant hereditary diseases caused by pathogenic germline variants in specified genes, and characterised by a broad spectrum of malignancies. Whereas HDGC is associated with *CDH1* and *CTNNA1* variants and defined by an increased risk of diffuse gastric cancer and lobular breast cancer, Lynch syndrome results from alterations in mismatch repair genes, whose main manifestations include colorectal, endometrial, ovarian, breast, prostate, stomach, and urological tumours. Remarkably, a huge difference remains in the knowledge surrounding the molecular mechanisms that drive these disorders, and in current approaches for patient management. In fact, the HDGC narrative is still in its early stages when compared with Lynch syndrome, which accumulates more than a century of research. Herein, we propose an analogy between HDGC and Lynch syndromes, highlighting intricacies across genetic origin, variant effects, cellular landscapes, and associated clinical outcomes. Further, we postulate that the history of Lynch syndrome may be useful to advance HDGC aetiology, namely strategies for identification of new candidate genes, rules for variant interpretation, sources of phenotypic heterogeneity, and improved surveillance protocols. This collected data will impact clinical perspectives, as well as future research programs addressing HDGC unmet challenges.

## Introduction

Hereditary cancer syndromes account for 5–10% of all cancers. Although this appears as a modest fraction, an incredible effort has been set to identify at-risk individuals carrying variants in cancer predisposing genes and who might benefit from enhanced surveillance and/or risk reduction measures. More so, uncovering a carrier of a causative variant elicits a cascade of genetic testing among family members to guide clinical management of susceptible individuals [1].

Hereditary diffuse gastric cancer (HDGC), also known as diffuse gastric and lobular breast cancer syndrome (DGLBCS), and Lynch syndromes are both dominant hereditary diseases caused by pathogenic germline variants in “specific genes” and characterised by a panel of malignancies [2, 3].

Originally described in 1998, in a New Zealand Maori family, HDGC is mostly associated with inactivating germline variants in the *CDH1* tumour suppressor gene, with pathogenic variants in *CTNNA1* also described in a minority of families [4, 5]. This syndrome is characterised by a significant increase in the lifetime risk of diffuse gastric cancer and lobular breast cancer, though other disease entities such as cleft lip/palate (CL/P) have been described in affected families [6, 7]. In the past decade, significant advances in next-generation sequencing technologies have expanded our capacity to identify patients at-risk, while highlighting our lack of knowledge regarding mechanisms that drive HDGC and effective approaches for patient management [8]. Further, increased genetic testing has resulted in the identification of *CDH1* variants associated with other clinical conditions unrelated with HDGC, including the Blepharocheilodontic syndrome, of which cleft lip/palate is a main manifestation [9].

Lynch syndrome, also known as hereditary non-polyposis colorectal cancer, was one of the first cancer predisposition syndromes to be described and, as a result of more than a century of multidisciplinary discoveries, our current understanding of this condition represents a role model for studying other hereditary cancer syndromes [10]. In fact, following the first documentation in 1895 of a family pedigree aggregating colorectum, stomach and uterus cancers, the term “Lynch syndrome” was coined in 1984 to describe an autosomal dominant condition characterised by a significantly increased risk of developing a spectrum of cancers that includes colorectal, endometrial, ovarian, small bowel, stomach, hepatobiliary, urinary, brain or central nervous system cancers, as well as sebaceous tumours [3]. It is estimated that Lynch syndrome accounts for approximately 3% of all diagnosed colorectal cancers [10].

The aetiology of Lynch syndrome is attributed to germline alterations in mismatch repair (MMR) genes that lead to microsatellite instability (MSI) [11]. Indeed, the primary diagnostic strategy for Lynch syndrome relies on germline testing for MMR gene variants in patients harbouring tumours with high-level of MSI and/or deficient MMR protein expression [3].

Herein, we provide an overview of the genetic landscape, molecular players and modifiers underlying HDGC and Lynch syndromes. We outline challenges regarding variant classification, as well as management and surveillance protocols that are common to both syndromes. The comparison does not aim to imply any molecular or mechanistic overlap but rather, the analogy is drawn at the conceptual level, emphasising how lessons learned from Lynch syndrome can inform similar approaches in HDGC.

## Genetic cause

Over the years, several studies have identified specific genes that, when mutated, promote uncontrolled cell growth and invasion of malignant cells [12]. It is widely accepted that acquired variants in somatic cells cause most of the sporadic cancers, while germline variants explain rare hereditary syndromes [13]. Moreover, cancer-related genes can be categorised into oncogenes and tumour suppressor genes. Oncogenes are phenotypically dominant, with the activation of a single mutated copy (allele) of a proto-oncogene being sufficient to promote cancer [13]. In contrast, tumour suppressor genes are phenotypically recessive, where deletion or inactivation of both alleles is necessary to impair gene function and cause cancer [13]. Inherited cancer syndromes often result from loss of function of tumour suppressor genes rather than from oncogene activation. In particular, HDGC [OMIM #137215] and Lynch syndromes [OMIM #120435] are the most common forms of hereditary gastric and colorectal cancer, respectively, and present an autosomal-dominant inheritance pattern [14]. Since mutations are inherited from one parent, every cell from the progeny carries a functional and a defective copy of the gene. Complete gene inactivation occurs upon somatic loss of the second allele (Knudson’s two-hit hypothesis) through additional mutation, hypermethylation, or other silencing mechanism [15, 16].

**Fig. 1:**
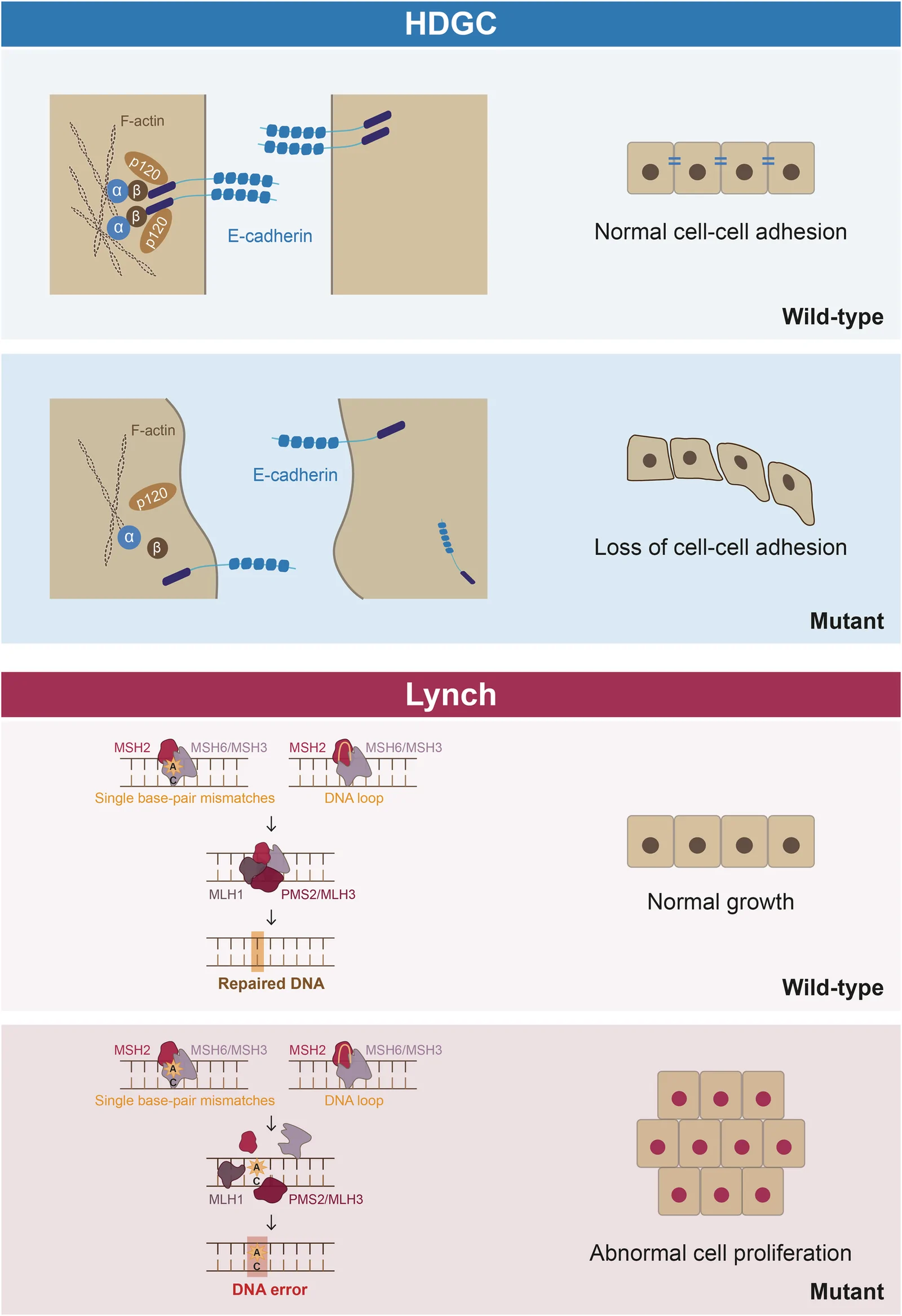
Cellular mechanisms underlying HDGC and Lynch syndromes. Schematic representation of the main molecules and corresponding functions involved in the aetiology of both cancer syndromes. E-cadherin and α-catenin are critical players for cell-cell adhesion, contributing to structural and mechanical integrity of normal epithelial tissues. HDGC arises as a consequence of germline variants in *CDH1* and *CTNNA1*, which encode E-cadherin and α-catenin, impacting cell-cell adhesion and invasion capacity. MMR genes *MLH1*, *MSH2*, *MSH6*, and *PMS2* code for proteins that assemble to recognise and repair DNA replication errors (wild-type context). In Lynch syndrome, MMR deficiency leads to accumulation of DNA errors and abnormal cell proliferation.

*CDH1* (OMIM #192090) and *CTNNA1* (OMIM #116805) are the only proven genes implicated in HDGC [17, 18]. *CDH1* is located on chromosome 16q22.1 and encodes for epithelial cadherin (E-cadherin). E-cadherin is a 120 kDa glycoprotein that comprises three major portions: an extracellular domain responsible for homophilic binding of E-cadherin molecules on adjacent cells; a transmembrane domain that functions as an anchor and stabiliser; and the intracellular portion that mediates linkage between E-cadherin and the cytoskeleton [19]. The *CTNNA1* gene is mapped into chromosome 5q31.2 and codes for α-catenin, one of the cytoplasmic binding partners of E-cadherin [20]. In fact, the cytoplasmic domain of E-cadherin attaches to different proteins, namely α-, β-, and p120-catenins, forming a cadherin-catenin complex. These binding partners regulate E-cadherin trafficking and activity at the plasma membrane, by interacting with signalling moieties, as well as with distinct cytoskeletal components [19]. In particular, α-catenin displays mechanosensory properties and controls a dynamic interplay with filamentous actin (F-actin), improving the recognition of intercellular cadherin bonds [21, 22]. E-cadherin and α-catenin play an indisputable role in cell-cell adhesion, contributing to tissue architecture and integrity (Fig. 1) [19]. More so, E-cadherin is a critical player in the orientation of the mitotic spindle in the epithelium, ensuring planar cell divisions and epithelial polarity [23]. Accordingly, an elegant model involving the misalignment of the mitotic spindle has been proposed as a mechanism underlying the initial steps of diffuse gastric cancer [8, 24].

Underlying the aetiology of Lynch syndrome is a set of genes involved in DNA MMR: *MLH1* (OMIM #120436), *MSH2* (OMIM #609309), *MSH6* (OMIM #600678), *PMS2* (OMIM #600259), and *EPCAM* (OMIM #185535) [3]. The MMR system corrects DNA errors occurring during the process of DNA replication, assuring fidelity of the daughter DNA strand and thus tissue homeostasis [25].

*MLH1*, *MSH2*, *MSH6*, and *PMS2* genes code for proteins that interact as heterodimers. Specifically, MLH1 pairs with PMS2, PMS1 or MLH3 to form MutLα, MutLβ or MutLγ complexes, whereas MSH2 couples with MSH6 or MSH3, forming MutSα or MutSβ complexes [25]. MutSα or MutSβ complexes recognise single base-pair mismatches and small insertions or deletion loops of DNA that result from replication errors of repetitive sequences – the so-called microsatellite regions [25]. MutLα, MutLβ or MutLγ promote the excision and resynthesis of DNA. It is thus not surprising that defects in MMR machinery cause an accumulation of DNA errors, originating MSI [25]. Importantly, depending on the affected gene, the extent of MMR deficiency is different and results in distinguishable phenotypes. For instance, *MLH1* or *MSH2* variants are associated with complete inactivation of MMR and high levels of MSI (MSI-H phenotype). In contrast, *MSH6* alterations cause partial MMR deficiency and, accordingly, a MSI-low phenotype can be observed [25, 26]. Of note, pathogenic germline variants in *MLH1* and *MSH2* account for the majority of clinically diagnosed cases, although estimates of the population prevalence pinpoint these alterations as the less prevalent among MMR variants [27, 28]. *MSH6* and *PMS2* variants, which are in fact more prevalent in the population, are identified as causative of Lynch syndrome in a minority of cases and are often underdiagnosed due to their lower penetrance and later age of onset [27, 28]. Defects in *EPCAM*, that lead to loss of *MSH2* expression by epigenetic inactivation, are even rarer [26, 29, 30].

Overall, it is clear that HDGC and Lynch syndromes are caused by deregulation of two unique cellular mechanisms – cell-cell adhesion and MMR function – which suggests that alterations in other genes from those specific pathways may underlie cases that remain genetically unexplained. Despite comprehensive germline and tumour testing, no pathogenic variant in the currently known genes have been identified in 60-70% of clinically defined HDGC cases [8], and in 10-15% of cases with suspected Lynch syndrome [31, 32].

## Variant landscape

The frequencies of *CDH1* variant types may vary depending on the compliance with clinical criteria for genetic screening in the HDGC context. A systematic review collected and analysed all published studies describing *CDH1* gene variants in gastric cancer patients from both high- and low-prevalence countries to understand how these alterations appear across different populations and study designs [33]. Selected studies were classified into three categories: series studies, which included multiple gastric cancer patients not necessarily tested according to specific criteria; family studies, where patients were tested based on the clinical criteria established by the International Gastric Cancer Linkage Consortium (IGCLC); and unknown studies, which included individuals lacking familial information regarding cancer phenotypes. This comprehensive evaluation revealed that missense variants represent the largest subset, with a frequency of 28.9%, in individuals affected by gastric cancer without compliance with criteria [33]. Deletions were detected in 24.6% of the cases, splice-site in 21.9%, and non-sense alterations in 19.3%. Insertions are less frequently identified, constituting 4.8% of all *CDH1* variants detected [33]. In contrast, screening in families fulfilling HDGC criteria shows the highest frequency of deletions (31.6%) and non-sense alterations (27.4%), followed by missense (16.8%), splice-site (15.9%), and insertions (8%) [33]. This shift in frequencies reflects lower penetrance of missense variants and, consequently, limited recognition of HDGC manifestations [34].

The effects of missense variants are often mild, when compared with truncating variants, due to the presence of a mutated protein with potential residual function, thus masking disease penetrance. In the case of truncated mRNA *CDH1* transcripts, it is established that nonsense mediated decay mechanisms are activated, leading to their downregulation and disease development [35].

With regard to *CTNNA1*, a few number of germline alterations have been reported in HDGC families, comprising frameshift, nonsense, and missense variants [5, 17, 18, 36]. While first estimates defined a 49–57% cumulative risk of diffuse gastric cancer in *CTNNA1* carriers at 80 years [37], this may represent an overestimation. More recent evidence supports that, in the context of HDGC, *CTNNA1* is best characterised as a moderate-penetrance gene, rather than conferring the very high risks initially suggested [38]. A large-scale study including 38112 individuals, ascertained on the basis of personal or family history of cancer, compared outcomes in *CTNNA1* and *CDH1* variant carriers. Among the 270 individuals carrying *CTNNA1* truncating variants, the observed prevalence was 2.6% for unspecified gastric cancer (7/270) and 3.7% for lobular breast cancer (10/270) [38]. These findings provide a more balanced picture of the cancer risk in *CTNNA1* carriers, although they also underscore that robust, prospective studies are still needed to accurately quantify lifetime risks.

Reflecting long term efforts and dedicated research to identify genetic factors associated with Lynch syndrome, the number of germline variants reported affecting MMR genes is currently higher than 3000. Among these alterations, approximately 40% affect *MLH1*, 34% *MSH2*, 18% occur in *MSH6* and 8% in *PMS2*, with variable distribution of variant types [26, 29, 30]. For instance, *MLH1* variants comprise 40% nonsense or frameshift, 40% missense, 11% splice-site, 7% large rearrangements, and 2% in-frame alterations [26]. *MSH2* variants have a similar distribution pattern with 49% of variants classified as nonsense or frameshift, 31% missense, 10% large rearrangements, 8% splice-site, and 2% in-frame [26]. In the case of *MSH6* variants, those include missense in 49% of cases, nonsense or frameshift in 43%, splice-site in 3%, in-frame in 3%, and large rearrangements in 2% [26]. Strikingly, *PMS2* presents the highest proportion of missense variants - 62%, followed by 24% of nonsense or frameshift, 10% of large rearrangements, 3% of splice-site and 1% of in-frame alterations [26]. Of note, penetrance of *MSH6* and *PMS2* variants is reduced, as verified by higher average age of onset, when compared with carriers of *MLH1* or *MSH2* variants [3, 26]. This might be associated with the higher frequency of missense alterations in *MSH6* and *PMS2* than in *MLH1* or *MSH2*.

Common to both cancer syndromes is the lack of variant hotspots, since alterations span the full coding sequence of all specified genes. Such feature can be attributed to the loss of function of tumour suppressor genes, whose effects can vary in penetrance and residual activity, but often converging on a similar final outcome. In addition, no definite correlations can be established between variant type and functional domains affected with clinical phenotypes. Despite that in a large multicentre study with 854 families no pathogenic/likely pathogenic missense variants could be associated with HDGC phenotypes [39], it has been verified that several other missense variants segregate with disease and are supported by functional data and histopathological findings [40, 41]. Concerning variant location, some studies claim to have defined a correlation with specific clinical presentations, although based in limited information in extent or scope [42].

## Variant interpretation

A major challenge in cancer syndromes is the accurate risk prediction for developing the disease, in order to provide patients with adequate surveillance and management schemes [5, 10]. In this setting, efforts have been made to implement variant classification systems discriminating potential pathogenic from benign genetic alterations. Variants that disrupt gene sequence, such as nonsense or frameshift, are more frequently identified as risk-conferring, while most benign variants are intronic, missense and splice site alterations. Remarkably, 28% of variants affecting *CDH1*, 31% for *MLH1*, 28% for *MSH2*, 47% for *MSH6*, and 26% for *PMS2* remain without a definite resolution and are classified as variants of uncertain significance (VUS), of which non-synonymous missense variants represent a large fraction, if not the largest [43, 44].

Currently, classification of variants occurring in MMR genes takes into account clinical and familial data, variant recurrence, allele frequency in control reference groups, in silico prediction algorithms, and studies on variant functional impact (InSiGHT Variant Interpretation Committee: Mismatch Repair Gene Variant Classification Criteria - Version 2.4 June 2018 https://www.insight-group.org/content/uploads/2018/08/2018-06_InSiGHT_VIC_v2.4.pdf). Irrespective of the difficulties that an integrative approach can raise, clinicians and genetic counsellors are committed to achieve a rigorous categorisation for better patient care.

Classification of *CDH1* variants is still immature due to a persisting conservative view that neglects high-quality scientific evidence and advances in experimental models [44]. Moreover, specific classification guidelines are not available for *CTNNA1* variants given the limited data available and moderate cancer risk verified in the context of HDGC [38]. Indeed, not disregarding that the molecular effects of variants in MMR and adhesion complex genes are distinct, a huge difference remains among the pipelines developed to access the functional impact of variants affecting MMR or *CDH1* genes. The effects of *CDH1* variants have been investigated through transfected cell lines and a combination of methodologies, which quantitatively evaluate the levels of protein yield, its localization, adhesive and anti-invasive functions, as well as cellular morphology and organisation [34, 40, 45]. These assays are low throughput and time-consuming, and are performed on a research-based standpoint, which is not compatible with current demands upon genetic panel testing. A simpler approach is settled in the case of MMR gene variants, encompassing examination of protein levels and MMR activity [46]. Whereas the deleterious outcome of missense variants leading to lower protein levels, as a result of their structural destabilization, is well-established in Lynch syndrome [47, 48], this effect is not considered in *CDH1* missense variants regardless of the vast number of studies showing similar phenotypes [40, 49, 50]. Likewise, a significant decrease in DNA repair efficiency tested in a cell-free system is sufficient to classify an MMR variant as “likely pathogenic” [51], while robust cellular assays showing loss of adhesive and anti-invasive functions of E-cadherin do not change variant classification. As an example, Figueiredo et al. have recently addressed the significance of the *CDH1* G212E variant identified in an unusually large pedigree displaying strong aggregation of diffuse gastric cancer [40]. The group has explored clinical observations, histological findings, in silico predictions, in vitro assays and in vivo strategies – all validating the deleterious nature of the genetic alteration [40]. Of note, an innovative *Drosophila* model was developed, which demonstrated that the G212E variant disrupts epithelial architecture and tissue organisation in vivo [40]. To date no *CDH1* missense variants have been recognised as disease-causing by the *CDH1* Variant Curation Expert Panel (VCEP) from the Clinical Genome Resource (ClinGen) [44]. In contrast, missense variant effects have been suggested to be mediated through impact on splicing, despite being based on software predictions, often in the absence of experimental validity (using minigene or additional RNA assays) and sequencing of expected alternative transcripts [44].

The controversy surrounding missense type variants spans a large panel of genes and clinical contexts, including those of other cancer syndromes such as Hereditary Breast and Ovarian Cancer (HBOC) [52], or inherited conditions like Cystic Fibrosis [53] and Familial Partial Lipodystrophy [54]. This lack of consensus is mainly due to incomplete penetrance of associated disease phenotypes, variability on family presentation, and residual protein activity, which impair a straightforward translation of functional defects into evident clinical outcomes. Mounting evidence highlights the need to develop research programs aiming to disclose the paradigm surrounding VUS and the role of other determinants impacting variant effects. In this sense, novel experimental approaches would provide additional indication of variant functional outcomes and improve dedicated classification schemes. For instance, *CDH1*-specific mutant cell lines could be engineered and cultured in 3D systems for subsequent monitoring of cell division orientation and spheroid organisation. Previous studies using *CDH1*-knockout organoid models have already demonstrated that complete loss of E-cadherin alters epithelial organisation and mitotic spindle dynamics, providing important mechanistic insight into HDGC pathogenesis [24, 55]. However, knockout systems represent total loss of function, and thus a homogeneous phenotype. Engineering organoids carrying specific *CDH1* variants, particularly of the missense type, could be viewed as complementary tools for the functional validation of clinically ambiguous variants, addressing a wider range of biological outputs, including partial protein destabilization or altered adhesive interactions with relevant partners.

## Disease phenotypes

HDGC and Lynch syndromes exemplify how germline variants can exert context-dependent effects on tumour development across different tissues. Although the mechanisms underlying tissue susceptibility remain largely unknown, contributing factors may include local gene expression and function, activation of downstream signalling pathways, and features of the tissue microenvironment.

**Fig. 2:**
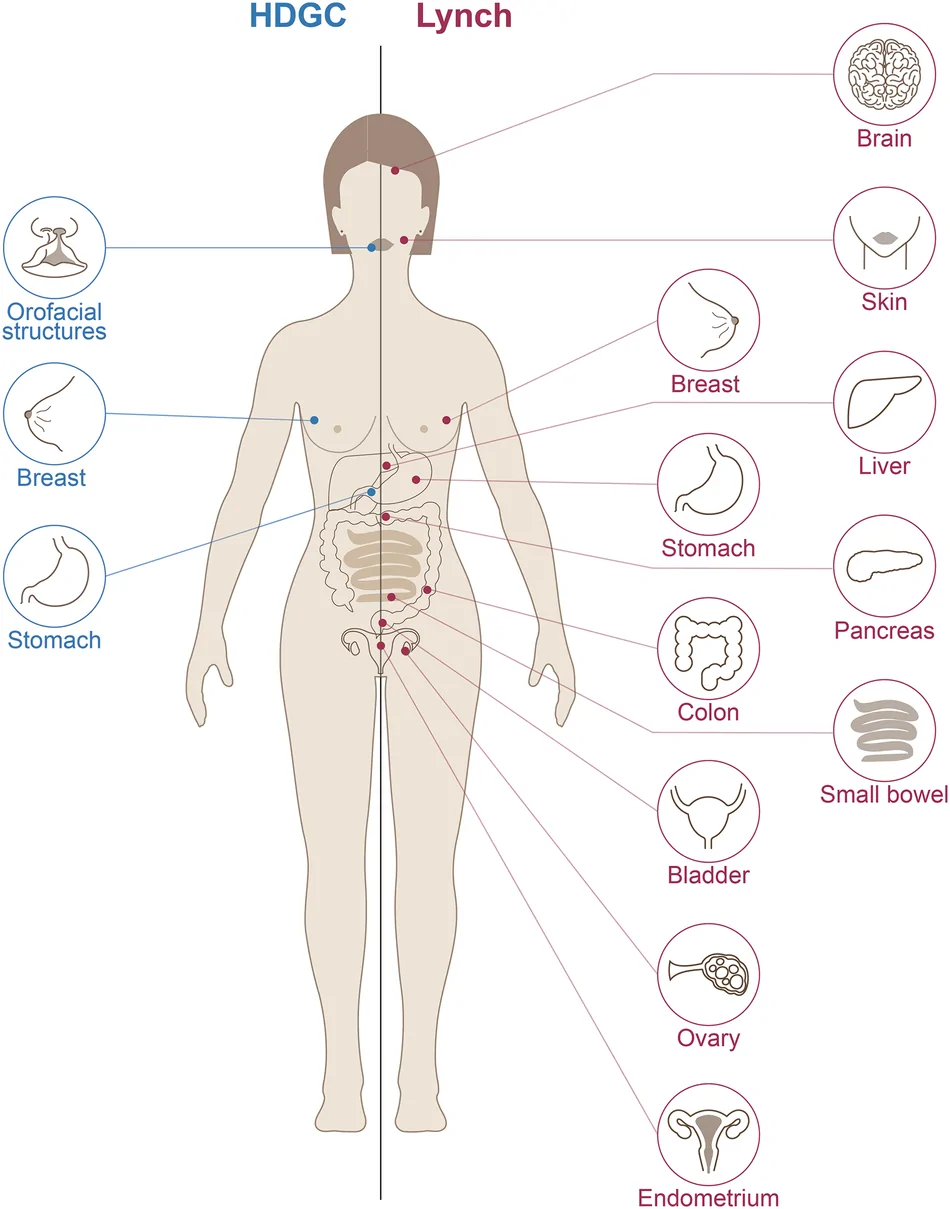
Clinical phenotypes associated with HDGC and Lynch syndromes. Disorders described in HDGC context comprise diffuse gastric cancer, lobular breast cancer and orofacial clefts (cleft lip and palate). Lynch syndrome is associated with a large panel of cancer types. These include colorectal, endometrium, prostate, breast, ovarian, urological tract (bladder, ureter or kidney), stomach, pancreas, brain (usually glioblastomas), small bowel, hepatobiliary tract, and skin tumours.

The tumour spectrum associated with Lynch syndrome has evolved over time. Cancers of the colorectum and endometrium were the first manifestations documented, with the publication of family G by Warthin in 1913. Increased frequencies of cancers of the stomach, small bowel, hepatobiliary system, upper urologic tract and ovary were reported 80 years later, in 1994 [56]. A study in 2008 added glioblastomas to the tumour spectrum [57], and only recently cancers of the pancreas, breast, and prostate, as well as the rare adrenocortical tumours, were considered to be phenotypic expressions of the Lynch syndrome [58–60]. Reliable associations between affected genes and clinical presentations have been observed for the broad spectrum of cancer types in Lynch syndrome (Fig. 2). Carriers of *MLH1* alterations have increased lifetime risk of developing colorectal (46%) and endometrium (43%) cancers, followed by prostate (17%), breast (12%), and ovarian (10%) neoplasms [61]. Cancers affecting urological tract (bladder, ureter or kidney, 8%), stomach (7%), and pancreas (6%) can also occur although at lower frequencies [61]. In the case of *MSH2* variants, endometrial (57%), colorectal (43%), prostate (32%), and urological tract (25%) cancers constitute the most frequent presentations, whereas *MSH6* loss of function confers higher risk for endometrium (47%) and lower risk for prostate (18%), colorectal (15%), ovarian (13%), breast (13%), urinary tract (11%), gastric (5%), and pancreatic cancers (1%) [61]. Concerning *PMS2* variants, major risks were reported for breast (56%), prostate (38%) and endometrium (26%) cancers [61], but according to recent studies these were not confirmed and only a marginal increase is detected, when compared with risks for the general population [28]. In addition, Lynch syndrome-associated tumours include those of the brain (usually glioblastomas), small bowel, hepatobiliary tract and skin (sebaceous tumours) [10, 62].

So far, a restricted panel of disorders has been described in HDGC patients. These comprise diffuse gastric cancer, which occurs in up to 70–80% of HDGC cases, with lifetime risks varying from 25% to 33% for women and 37% to 42% for men, and a mean age at onset of 45 years [63, 64]. This contrasts with Lynch syndrome, where gastric cancer represents only a minor fraction of the clinical presentations, underscoring the substantial differences in tissue-specific cancer risk between the two syndromes. Likewise, lobular breast cancer is highly prevalent in HDGC, accounting for the second most frequent neoplasia in 39% to 55% of female variant carriers, with a mean age at onset of 48–53 years [63, 64]. Importantly, among families from North America with germline *CDH1* pathogenic/likely pathogenic variants, the cumulative risk of gastric cancer was recently conveyed as only 7% to 10%, whereas the cumulative risk of breast cancer in female carriers remained similar to prior estimates [65].

HDGC kindreds have also been reported to display cases of colorectal cancer. In fact, colorectal cancer with signet ring cell features appears to be enriched in patients harbouring pathogenic or likely pathogenic *CDH1* variants, but the number of cases identified in HDGC families are scarce [4, 66, 67]. Moreover, data suggest that the lifetime risk of invasive colorectal cancer in HDGC may be similar to that of the general population, hampering its inclusion within the HDGC disease spectrum [66, 68]. Congenital malformations, like orofacial clefts, have also been associated with HDGC [6, 7], and in accordance, occurrence of cleft lip, cleft lip and palate, or cleft palate alone now constitutes a criterion for *CDH1* genetic screening [5]. Of note, phenotype presentation varies largely among families as these may display diffuse gastric cancer only or combined with the abovementioned manifestations.

*CDH1* germline variants are not restricted to the classical HDGC spectrum since individuals who do not meet the clinical criteria for HDGC may still be at risk of hereditary lobular breast cancer (HLBC). The study by García-Peláez et al. demonstrated that 7% of pathogenic/likely pathogenic variants were detected in families presenting only lobular breast cancer, not fulfilling the 2020 HDGC criteria [39]. Similarly, Corso et al. conducted a large longitudinal study with 5429 lobular breast cancer patients, revealing that HLBC represents 34.4% of cases. Multigene panel testing of 394 cases identified *CDH1* pathogenic/likely pathogenic variants in 1.5% of patients with early-onset disease and a family history of breast cancer [69].

It is clear that clinical presentations in both hereditary conditions differ even in individuals with similar genotypes, suggesting that other genetic and non-genetic factors modulate disease phenotypes. Considering the undisputable role of E-cadherin during embryonic development and in adult epithelia, it is not unexpected that the range of cancers, as well as congenital malformations, arising in HDGC context may expand in the upcoming years. Likewise, we postulate that a plausible profile of disorders may emerge depending on the disturbed gene.

## Management and surveillance protocols

As described above, individuals with inherited cancer syndromes have increased susceptibility to develop tissue-specific cancers at an early age. Therefore, a successful outcome and improved overall survival is dependent on long term and adequate management protocols at specialised centres. While proper surveillance guidelines are already at place for some hereditary cancer syndromes, comprehensive recommendation protocols are still underdeveloped for others.

Lynch syndrome is a valuable example of integrative patient management, in view of its association with an increased lifetime risk of developing many cancer types. Improvements in patient follow-up procedures have indeed accompanied the emergence of new data, mostly due to technological advances and accurate identification of susceptible individuals. Patients diagnosed with Lynch syndrome are being offered coordinated multidisciplinary support, strongly reducing the risk of developing cancer and impacting their quality of life. Clinical surveillance and intervention guidelines differ in screening starting age and intervals, depending on the affected gene (*MLH1*, *MSH2*, *MSH6* or *PMS2*) and the proposing entity [3, 70, 71]. In particular, for patients with *MLH1* or *MSH2* variants, the National Comprehensive Cancer Network (NCCN, United States) suggests screening should start at 20-25 years for colorectal, 30-35 years for gynaecological and urological, and 40 years for gastroduodenal cancer if associated with risk factors [3]. In these cases, screening intervals range from 1-2 years for colorectal and gynaecological, and 3-5 years in gastroduodenal surveillance. There are no recommendations for urological screening interval. According to the observed lower risk for colorectal cancer associated with *MSH6* or *PMS2* variants, screening should start at a later age (30-35 years) and be maintained at intervals of 1-3 years [3]. In contrast, European guidelines are less strict and suggest colorectal surveillance should initiate at the age of 25 years for *MLH1* or *MSH2* variant carriers, or 35 years for *MSH6* or *PMS2* variants. Screening intervals range from 2-3 years for *MHL1*, *MSH2*, and *MSH6* variants or 3-5 for *PMS2* [3, 70]. Likewise, urological surveillance should start at 40-50 years and should comprise urinalysis, urine cytology, and abdominal ultrasound every 2 years for *MLH1*, *MSH6,* and *PMS2* variants, or yearly for *MSH2* variants [72]. Notably, no surveillance is recommended for gastroduodenal lesions, whereas for gynaecological it is offered an optional annual review starting at the age of 25 years [70, 73]. In addition to these, risk-reducing colon and gynaecological surgeries, as well as aspirin intake can be considered as part of the NCCN and European guidelines [70, 71]. Clinical management encompassing endoscopy, screening for *H. pylori* infection, and assessment of physical/neurological capabilities have also been suggested [70]. Nonetheless, the potential benefits of the latest remain unclear and are not performed on a regular basis [74]. To ameliorate the identification of individuals with Lynch syndrome, genetic testing has been expanded and is now proposed for all patients diagnosed with colorectal cancer before the age of 50 years [11, 70].

Multidisciplinary surveillance in the context of HDGC is still in its infancy. Diffuse gastric cancer and lobular breast cancer are undoubtedly the main cancer presentations in HDGC, although with variable penetrance. According to current guidelines, genetic testing for HDGC should be offered from the legal age of consent (generally 16-18 years) in individuals meeting testing criteria, while testing of younger family members may be considered when warranted by family history. Clinical management of *CDH1* carriers involves intense endoscopic surveillance and/or prophylactic gastrectomy, as well as mammography and magnetic resonance imaging screening [5]. Specifically, for carriers of pathogenic/likely pathogenic *CDH1* variants with confirmed cases of diffuse gastric cancer in first- or second-degree relatives, prophylactic total gastrectomy is advised [5]. Alternatively, carriers who have decided to undergo surveillance, should perform annual endoscopy at specialised centres, with IGCLC guidelines recommending extensive analysis of 30 random biopsies along the cardia, fundus, body, transition zone, and antrum, as well as targeted biopsies of visible abnormalities [5]. Asif et al. have provided evidence highlighting the critical role of targeted biopsies in HDGC surveillance [75]. In a prospective study, the authors demonstrated that targeted sampling of subtle mucosal changes substantially improved the detection of early signet-ring cell carcinoma, emphasising the need for combining both approaches in endoscopic protocols [75]. Indeed, the presence of latent or transient intramucosal signet-ring cell carcinoma foci are pathognomonic of HDGC with no equivalent in Lynch syndrome. Early immune changes within the gastric mucosa of *CDH1* variant carriers may promote signet-ring cell dormancy, justifying the persistence of latent foci and their variable progression to invasive cancer [76].

As for individuals with *CDH1* VUS, the IGCLC recommends annual endoscopic surveillance for at least 2 years. After this period, the interval may be increased [5]. It is important to note that counselling of these patients should balance the burden of gastric cancer risk and the psychological impact of prophylactic surgery and intense surveillance [5]. NCCN guidelines propose a more balanced approach, placing endoscopic surveillance on equal footing with prophylactic gastrectomy. This reflects emerging data on the potential safety of surveillance strategies, and the recognised risks and quality-of-life impact associated with prophylactic gastrectomy [77].

Considering breast cancer, it is recommended that women with a pathogenic/likely pathogenic *CDH1* variant undergo annual surveillance from 30 years of age, which includes annual magnetic resonance imaging possibly supplemented with mammography, starting at 35-40 years of age [5]. Bilateral risk-reducing mastectomy can also be considered in a case-by-case scenario [5]. In addition to diffuse gastric cancer and lobular breast cancer, other cancer types and congenital disorders have been observed in HDGC kindred, although at a much lower incidence [6, 7, 66]. In contrast to Lynch syndrome, these less frequent manifestations within the HDGC tumour spectrum are not yet considered for surveillance protocols in the guidelines since their incidence has been shown to be comparable to that of the general population [5]. We envisage that future research will provide robust data concerning risk estimates of other cancers and congenital manifestations within HDGC kindreds.

The evolution of genetic testing criteria and the trend towards a more liberal testing approach represent an emergent aspect of both Lynch syndrome and HDGC management. While initial testing strategies were highly restrictive, relying on stringent clinical and familial criteria, broader tumour screening and germline testing criteria have been adopted in Lynch syndrome, resulting in increased detection rates. A similar movement is now seen in HDGC, with recent NCCN guidelines recommending more inclusive criteria for genetic testing of *CDH1* and, more cautiously, of *CTNNA1*, overcoming potential dismissal of at-risk individuals [77].

## Conclusions

Compelling evidence demonstrates that recognition of clinical, pathological and molecular features of Lynch syndrome has evolved drastically since the first reports. Interestingly, it is noticeable that HDGC and Lynch syndromes are attributed to dysfunction of genes involved in two distinct but well-defined cellular processes, namely cell-cell adhesion and MMR system. Germline alterations are evenly distributed across the entire genes, without categorical correlations established between variant types or protein domains affected. Additional complexity incurs in light of missense variants due to residual protein activity and consequent lower penetrance of disease phenotypes.

We foresee that the intricacies and history of Lynch syndrome may serve as a paradigm to progress in HDGC identity. Indeed, the extended knowledge acquired within the scope of Lynch syndrome may unlock new avenues of research to elucidate novel causative genes, the significance of missense variants, determinants of tissue-specific effects, and environmental modulators of clinical presentations in HDGC. Single-cell multi-omics technologies providing information on cell functional states and spatial dynamics will be critical to advance the molecular profile of E-cadherin deficient cells and signalling programs underlying HDGC.
